# Affective and fatigue responses to incremental exercise: the effect of hypoxic and normoxic training protocols

**DOI:** 10.3389/fspor.2025.1702420

**Published:** 2026-01-12

**Authors:** Stela Pismiri, Nektarios A. M. Stavrou, Igor B. Mekjavic

**Affiliations:** 1 School of Physical Education & Sport Science, National & Kapodistrian University of Athens, Dafni, Greece; 2 Department of Automation, Biocybernetics and Robotics, Jozef Stefan Institute, Ljubljana, Slovenia

**Keywords:** emotional state, fatigue, hypoxia, mental mood, normoxia, training, V̇O_2max_

## Abstract

**Introduction:**

Hypoxic training protocols have been used to improve athletes' performance by influencing both physiological and emotional responses. The aim of the present study was two-fold. First, to evaluate the post-exercise emotional response on the increasing intensity exercise (O_2max_) in normoxia and hypoxia. Second, to assess the emotional responses of two hypoxic training protocols (Sleep High-Train Low, SH-TL; Sleep Low-Train High, SL-TH) during increasing intensity exercise (O_2max_) in both normoxia and hypoxia.

**Methods:**

Twenty-seven physically active males ranging in age from 18 to 30 years volunteered to participate in the study. The participants were randomly assigned into two different hypoxic training protocols (SH-TL, SL-TH), and a control group. Participants performed a protocol to evaluate O_2max_ in hypoxia and in normoxia. The emotional responses were evaluated based the Activation Deactivation Adjective Check List and the Multidimensional Fatigue Inventory. Repeated measure analysis of variance was applied to examine the differences on participants' mood state and fatigue (energy, tiredness, tension, calmness, general fatigue, physical fatigue, reduced activity, reduced motivation, emotional fatigue) before and after the of O_2max_ before (pre-test), in the middle (middle-test), and in the end (post-test) of each training protocol.

**Results:**

A significant change in energy in the SL-TH after O_2max_ was revealed compared to the other groups, between pre- and post-test. In the SH-TL condition, significant differences were found in energy and tension in normoxia, and in tension in hypoxia conditions. In the SL-TH group, significant differences were indicated in tiredness and calmness in normoxia. In hypoxic condition, significant changes were found in tiredness, tension, calmness and general fatigue.

**Discussion:**

These findings point towards the effectiveness of SL-TH in managing fatigue and maintaining positive emotions. The effect of hypoxic training was noticed in participants mood state, highlighting the importance of a proper design aiming to improve humans' performance in hypoxic and normoxic conditions.

## Introduction

Exposure to hypoxic conditions and various hypoxic training protocols have been employed to maximize performance in both normoxic and hypoxic environments in sports (1). Hypoxia creates an environment in which the human body and mind attempt to adapt (acclimatization) on a regular basis. Numerous studies have thoroughly investigated the effects of hypoxia on physiological responses (2–4). While several studies have examined psychological effects such as mood, behavior, and cognitive function, findings remain inconsistent, with some supporting negative psychological impacts and others reporting minimal or no effects (5–7). Despite this growing body of research, a specific gap persists: few studies have systematically compared how different hypoxic training protocols affect psychological outcomes -particularly mood and cognitive function- across both hypoxic and normoxic conditions. This lack of comparative data on protocol-specific effects limits our understanding of which approaches may best optimize psychological aspects of athletic performance. Thus, the key question is how varying hypoxic training methods differentially impact mood and cognition, especially during prolonged exposure. Addressing this knowledge gap is essential for refining hypoxic training strategies to enhance overall psychological readiness in athletes in both normoxic and hypoxic environments, contributing to a deeper understanding of the relationship between hypoxia and emotional adaptation. Exercise is well known to positively influence psychological responses by increasing positive emotions and reducing negative emotions (8) in hypoxic conditions. The positive impact of exercise on mood, affect, cognition, and brain activity is well-established (9), although the magnitude of the effect is highly dependent on exercise type, exercise intensity, and participant characteristics (e.g., age, physical condition) (10, 11).

Studies investigating psychological changes in response to hypoxia demonstrate a variety of impairments, including mood alterations, behavior changes, fatigue, shifts in motivation, impairments in cognitive function, and coordination issues (12, 13, 57). Field studies examining the effects of altitude on emotional responses tend to show that negative mood increases during hypoxia (14–21, 54). Specifically, there seems to be an evident increase during hypoxia on anger, confusion, fatigue, depression, and tension (22–24), while calmness and activation tend to decrease (25). However, other studies offer mixed findings. Hypoxia improve mood only after exercise of low to moderate intensity (40%-60% V̇O_2max_) [Seo, Fennell, Burns, Pollock, Gunstad, et al., 2015 (26, 27)]. Other studies report no negative effects on participants' emotions in hypoxic conditions (6, 23, 28, 29). These conflicting findings may be due to differences in exercise intensity, exposure duration, or participant characteristics such as age and fitness level. The variability shows we need to investigate how these variables affect emotional responses to exercise in hypoxic conditions. According to these studies, it remains unclear whether exercise-induced emotional responses are influenced by hypoxia and exercise.

Fatigue is a multifaceted concept characterized by emotional, behavioral, and cognitive alterations (30, 31). In addition to the symptoms mentioned previously, the feeling of fatigue seems to be significantly important during human exposure to a hypoxic environment. Research studies, however, have focused mainly on the physiological aspects of fatigue, although its psychological characteristics are of great interest and can significantly modulate individuals' operational and/or physical capacity. Fatigue is conceptualized in various ways (acute vs. chronic, physiological vs. psychological, central vs. peripheral), and is characterized by emotional, behavioral, and cognitive alterations (30, 31). In the current study, we adopted the concept of fatigue as a self-reported state mood construct of perceived fatigability, characterized by reduced motivation and cognitive function, as well as an individual's inability to perform mental tasks or physical activities, characterized by emotional conflict (58, 59). To assess fatigue, we employed the Multidimensional Fatigue Inventory (32) and the Activation Deactivation Adjective Check List (33), which are well-established instruments for evaluating various aspects of fatigue and mood. These tools provide a comprehensive approach to understanding fatigue from both psychological and physiological perspectives. The role and understanding of mental fatigue have been rarely investigated in a hypoxic environment. However, research findings support the notion that fatigue can significantly affect a person's decision-making, cognitive activity, psychological response, and physical performance (34–36). Virues-Ortega et al. (37) also suggest that fatigue may have a mediating or structuring role in psychological, mental, and cognitive performance during human exposure to a hypoxic environment.

The aim of the present study was to investigate the effect of different hypoxic training protocols (sleep high-train low, sleep low-train high) on exercise-induced psychological responses. These training protocols were chosen because they are two prominent methods in hypoxic training, each with distinct physiological and psychological implications. The “Sleep High-Train Low” protocol lets athletes be exposed to hypoxia during recovery. This can spur physiological adaptations but might also increase negative affect due to stress during rest, which may influence mood and motivation. In contrast, the “Sleep Low-Train High” approach focuses on maximizing high-intensity performance in hypoxia. This method might enhance psychological resilience and mood by promoting stress adaptation, which there is a need of further examination. An additional purpose was to investigate: (a) the post-exercise emotional response to increasing intensity exercise (V̇O_2max_) in normoxia and hypoxia, and (b) the emotional responses of two training protocols at increasing intensity exercise (V̇O_2max_) in normoxia and hypoxia. Based on the above, we hypothesized that the “Sleep High-Train Low” training protocol will lead to a significant increase in negative emotional responses compared to the “Sleep Low-Train High” protocol during V̇O_2max_ testing under both hypoxic and normoxic conditions. It is further expected that no substantial differences will be observed in positive emotional responses between the two protocols under both conditions. Finally, both protocols are anticipated to result in increased fatigue following the V̇O_2max_ test.

## Materials and methods

### Participants

The sample size determination was based on the following criteria: (1) effect size = 0.319 based on a similar examined variables, and experimental conditions followed by Keramidas et al. (25), (2) power (1–*β*) = 0.80, (3) *α* = 0.05, (4) three experimental groups (SH-TL, TL-SH, and control), and (5) three repeated measures (pre-, middle-, post-test). The analysis showed that the minimum sample for the specific study was 24 participants (Fall et al., 2007). Twenty-eight men invited to participate in the study, and one of the SH-TL group was excluded of the study because of drop out. Based on the above, twenty-seven healthy men participated in the study (age: 22.6 ± 3.5 years; body mass: 74.0 ± 6.3 kg; height: 179.9 ± 5.7 cm; body fat: 10.8 ± 3.3%) and were randomly assigned to one of three training protocols: Sleep Low-Train High group (*n* = 10; age: 20.40 ± 2.76 years; weight: 76.61 ± 8.56 kg; height: 182.31 ± 4.37 cm, BMI: 11.39 ± 4.00 kg/m²), Sleep High-Train Low group (*n* = 9; age: 23.67 ± 3.24 years; weight: 70.78 ± 4.92 kg; height: 178.78 ± 5.21 cm; BMI: 10.23 ± 1.66 kg/m²), and control group (*n* = 8; age: 24.75 ± 3.01 years; weight: 73.99 ± 3.83 kg; height: 176.94 ± 5.15 cm; BMI: 10.05 ± 2.27 kg/m²). All participants were healthy, with no history of cardiovascular, pulmonary, or psychiatric issues. All participants were residents near sea level and had not been exposed to an altitude of more than 500 meters for at least one month prior to their participation in the experiment. They were instructed to avoid strenuous exercise and to abstain from alcohol or caffeine consumption the day before the pre-, mid-, or post-tests. The study protocol applied was approved by the National Committee for Medical Ethics at the Ministry of Health of the Republic of Slovenia and was conducted in accordance with the principles outlined in the Declaration of Helsinki.

### Experimental design

The Sleep High-Train Low protocol involved daily exposure to simulated altitude for 9 h, progressively increasing from 2,800 m in the first week to 3,000 m in the second, and then stabilizing at 3,200 m during the final two weeks. Training was conducted under hypoxic conditions for 60 min per day at an intensity corresponding to 50% of the normoxic peak power output (PPO). In contrast, the Sleep Low-Train High protocol consisted of training under hypoxia with a fractional inspired oxygen concentration (FiO₂) of 0.12 (12% O₂), for 60 min daily at 50% of the hypoxic peak power output (PPO), while recovery occurred in normoxic conditions, allowing for physiological adaptation without additional hypoxic stimulus during rest.

**Figure 1:**
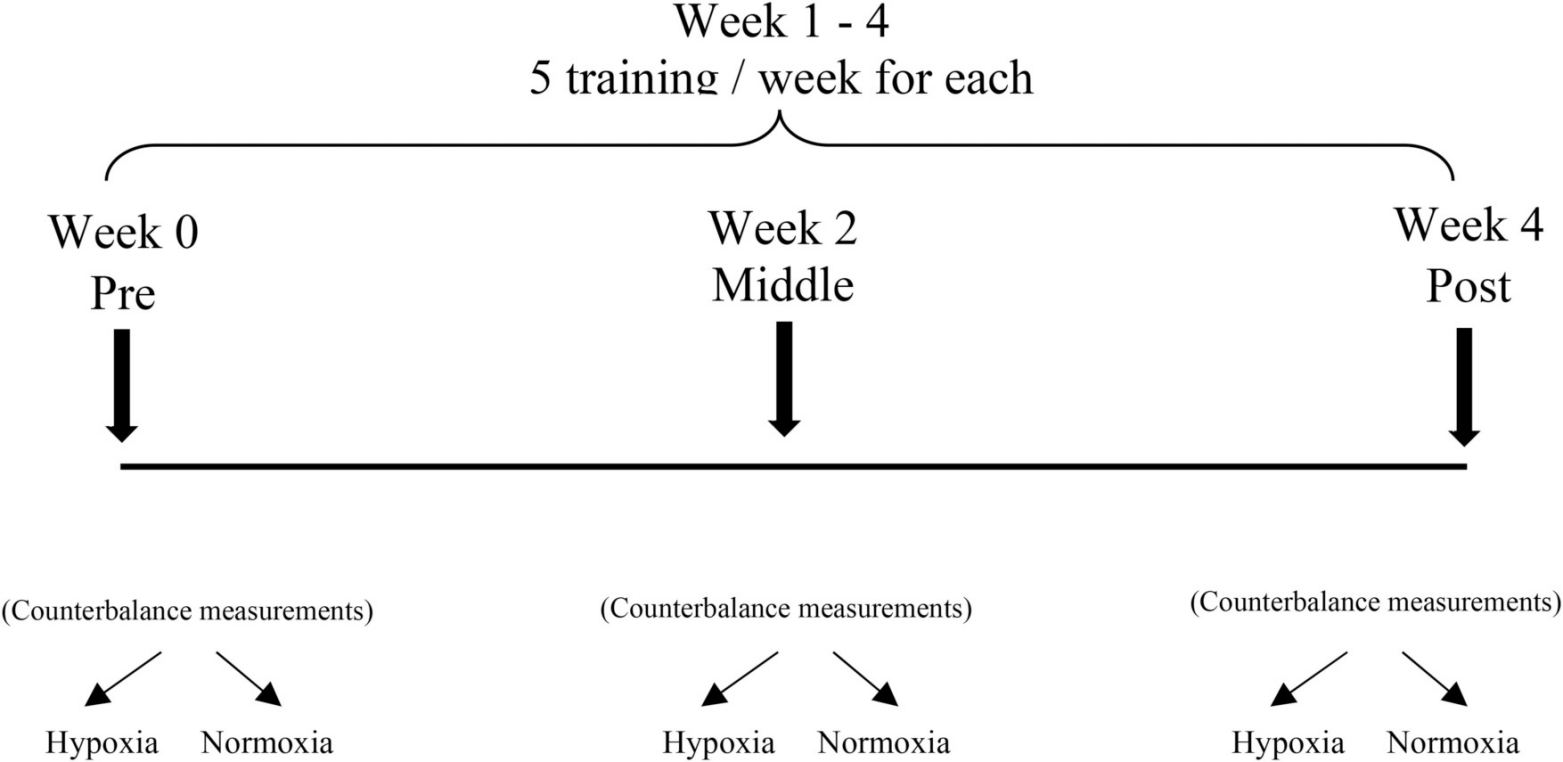
Research methodology outline.

The experimental protocol consisted of 20 training sessions on a cycle ergometer over 4 weeks (5 sessions per week) (Figure 1). Each training session included a 5-minute warm-up at 20% of normoxic peak power output (PPO), followed by a 60-minute exercise bout with a 5-minute rest period. Exercise intensity corresponded to the heart rate (HR) reached at 50% PPO, which was determined by the maximum aerobic test (V̇O_2max_) in normoxic or hypoxic conditions in a previous session. Therefore, the Sleep High-Train Low group and the control group participants trained at 50% of their normoxic PPO, while the Sleep Low-Train High group trained at 50% of their hypoxic PPO. The control and Sleep High-Train Low group participants performed all their training sessions in normobaric normoxia conditions, at 300 meters altitude (laboratory altitude). The Sleep Low-Train High group performed their training in a climatic chamber (IZR d.o.o., Skofja Loka, Slovenia) at the Jozef Stefan Institute, which maintained the temperature and humidity at 25 °C and 50%, respectively. The normobaric hypoxic environment (F_1_Ο_2_ = 0.12) was maintained with a Vacuum Pressure Swing Adsorption system (b-Cat, Tiel, The Netherlands) that supplied oxygen to the chamber. Samples of the chamber air were frequently analyzed for oxygen and carbon dioxide (CO_2_), and the oxygen delivery was adjusted based on the results of the gas analysis. If the oxygen level reached the specified fraction, oxygen delivery was stopped. If the oxygen level dropped below the preset level or the concentration of CO_2_ increased by 0.5%, a large industrial fan was operated, drawing normoxic air from the external environment into the chamber. Since gas was constantly flowing into the chamber, a relief valve prevented any excess pressure in the chamber room.

During the training sessions, the participants' HR was monitored (Hosand System, Verbania, Italy) and external workload was adjusted accordingly to maintain HR. The HR monitoring software was used to establish HR training sessions for each participant. The individual interval was defined as ± 4 bpm of the targeted HR measured during the first V̇O_2max_ test performed in normoxic (SH-TL and control group) or hypoxic (SL-TH) conditions, respectively. Whenever a participant's heart rate was outside the specified interval, the visual display prompted the researcher to increase or decrease the participant's activity rate.

Hypoxic and normoxic aerobic capacity was evaluated before (pre-test), in the middle (mid-test), and at the end (post-test) of the training protocols. Participants were instructed not to engage in any physical activity for at least 2 days prior to the tests, and to abstain from caffeinated beverages for at least 4 h prior to the performance test. Each test period (pre-, mid-, post-tests) consisted of 2 days of testing separated by a rest day. During the test days, daily performance tests were conducted: one incremental (V̇O_2max_) test to exhaustion in either normoxia or hypoxia. The sequence of hypoxic and normoxic tests was randomized and counterbalanced. Finally, anthropometric measurements were taken on different days before the day of the exercise test.

### Instrumentation

#### The activation-deactivation adjective checklist

The Activation Deactivation Adjective Check List [ADACL (33)] is a 20-item self-assessment tool and evaluates the energy, tension, tiredness, and calmness. The participants respond to each of the ADACL items based on how they feel. Each item is answered on a four-point scale, ranging from 1- “definitely do not feel” to 4-“definitely feel”. A score for each subscale was calculated by adding the scores of each item, with the total score ranging from 5 to 20.

#### Multidimensional fatigue inventory

The Multidimensional Fatigue Inventory [MFI (32)]. is a multidimensional self-rating instrument containing 20 items measuring five different aspects of fatigue: general fatigue, physical fatigue, reduced motivation, reduced activity, and mental fatigue. Each MFI subscale consists of four items, and the responses range from 1-“yes, that is true” to 5-“no, that is not true”. The values of each factor ranged from 4 to 20; the higher the value in each subscale represent participant's higher fatigue. The reliability and the validity of the MFI is well established.

### Procedure

The study was conducted at Valdoltra Orthopedic Hospital (Ankaran, Slovenia). During the first visit to the laboratory, participants were thoroughly familiarized with the equipment and the experimental procedure. Subsequently, they performed an incremental exercise test to exhaustion on an electric brake cycle ergometer (ERG 900S, Schiller, Baar, Switzerland). During the test, participants breathed either room air [AIR; fraction of inspired O_2_ (FiO_2_): 0.21] or a hypoxic gas mixture (HYPO; FiO_2_: 0.12; corresponding to a simulated altitude of approximately 4,500 meters). The trial was performed in a counter-balanced order, at the same time of day (morning), and within a 48-hour period to avoid any residual effects from the previous exercise test. Mean temperature, humidity, and barometric pressure in the laboratory were 22.2 ± 0.6 °C, 54 ± 6%, and 754 ± 5 mm Hg, respectively.

Each test commenced with a 10-minute rest period on the ergometer to establish baseline values. In the hypoxic condition test, the 10-minute rest period included 5 min of breathing oxygen, followed by 5 min of breathing hypoxic air. Participants were then instructed to perform 2 min of light work at a work rate of 60 W. Subsequently, the workload was increased by 30 W·min^−^¹ until exhaustion. Participants chose their preferred pedaling rate (between 60 and 70 rpm). The achievement of maximum oxygen consumption (V̇O_2_ peak), defined as the highest V̇O_2_ over 60 s, was determined according to the following criteria, listed in priority order: (i) exhaustion or severe fatigue causing an inability to maintain the given workload (cycling rate below 60 rpm), (ii) a V̇O_2_ plateau, and/or (iii) a subjective evaluation of exercise intensity at or near maximal. During the trial, participants remained seated on the ergometer and received constant verbal encouragement in a consistent manner from the same examiner. Before and immediately after each trial, participants were asked to complete two questionnaires.

Regarding the completion of the instruments, five minutes before and five minutes after each test, participants were asked to complete the Activation Deactivation Adjective Check List, based on how they felt during the time of completion. During the post-exercise phase, they also completed the Multidimensional Fatigue Inventory. All questionnaires were presented in a hardcopy format, and the researcher explained them to the participants before each trial. The subjects answered the questions in 4 to 8 min, sitting comfortably in a chair and breathing room air.

### Data analysis

A repeated-measures analysis of variance (ANOVA) was conducted to investigate the effects of exercise performed under normoxic and hypoxic conditions, both before and after V̇O_2max_, across two hypoxic training sessions (SH-TL and SL-TH), as well as in the control group, measured at two time points (pre- and post-intervention). Additionally, the variations in emotional responses were assessed across three time points (pre-, mid-, and post-intervention) for each hypoxic training protocol (SH-TL and SL-TH). Tukey’s *post-hoc* test was employed to identify significant differences revealed by the ANOVA. The partial eta square (*η*^2^_p_) was also estimated, as measures of the effect size for group mean differences (38). Tukey's corrected *t*-tests followed any significant between and within effects in the ANOVA models testing pairwise comparisons. Results are reported using a stricter threshold (*p* < .001) to reflect the strength of the evidence and reduce the risk of Type I error. The internal consistency of the ADACL and MFI subscales was examined with Cronbach's a coefficient. Cronbach's *a* value ranged for the ADACL from 0.64 to 0.83, and for the MFI subscales from 0.66 to 0.87, indicating an acceptable subscale reliability. Statistical analyses were performed using Statistical Package for the Social Sciences (SPSS) 26.0 (IBM Corporation, NY, USA).

## Results

Psychological results in post-exercise effects in normoxia and hypoxia before and after V̇O_2max_

### Activation deactivation adjective check list

The results of the repeated measures analysis indicated significant interaction only on energy after V̇O_2max_ in SL-TH participants (*F*_2,18_ = 4.982, *p* < .05, *η^2^p* = .237). The results showed significant decrease from pre- to post-test (*F*_2,18_ = 15.954, *p* < .001, *η^2^_p_* = .499), but no significant changes between conditions (*F*_2,18_ = .044, *ns*, *η^2^_p_* = .003). The energy before V̇O_2max_ didn't showed significant interaction (*F*_2,18_ = .338, *ns*, *η^2^_p_* = .021). No other significant interaction pointed out in SL-TH before and after V̇O_2max_ in tiredness (*F*_2,18_ = 2.308, *ns*, *η^2^_p_* = .126 - *F*_2,18_ = .749, *ns*, *η^2^_p_* = .045), tension (*F*_2,18_ = 1.702, *ns*, *η^2^_p_* = .096 - *F*_2,18_ = 3.482, *p* = .080, *η^2^_p_* = .179) and calmness (*F*_2,18_ = .188, *ns*, *η^2^_p_* = .012 - *F*_2,18_ = .906, *ns*, *η^2^_p_* = .054).

The results of the repeated measures analysis indicated a non-significant Time X Condition interaction before and after V̇O_2max_ in the ADACL subscales (energy, tiredness, tension, and calmness) in the SH-TL participants (energy: *F*_2,18_ = 2.208, *ns*, *η^2^_p_* = .121 - *F*_2,18_ = .835, *ns*, *η^2^_p_* = .053- tiredness: *F*_2,18_ = .007, *ns*, *η^2^_p_* = .000 - *F*_2,18_ = .153, *ns*, *η^2^_p_* = .010- tension: *F*_2,18_ = .000, *ns*, *η^2^_p_* = .000 - *F*_2,18_ = .026, *ns*, *η^2^_p_* = .002- calmness: *F*_2,18_ = .075, *ns*, *η^2^_p_* = .005 - *F*_2,18_ = 2.946, *ns*, *η^2^_p_* = .164).) and in control group (energy: *F*_2,18_ = .361, *ns*, *η^2^_p_* = .025 - *F*_2,18_ = 1.446, *ns*, *η^2^_p_* = .094 - tiredness: *F*_2,18_ = .709, *ns*, *η^2^_p_* = .048 - *F*_2,18_ = .069, *ns*, *η^2^_p_* = .005 - tension: *F*_2,18_ = .045, *ns*, *η^2^_p_* = .003 - *F*_2,18_ = 1.487, *ns*, *η^2^_p_* = .096 – calmness: *F*_2,18_ = .094, *ns*, *η^2^_p_* = .007 - *F*_2,18_ = 1.494, *ns*, *η^2^_p_* = .096).

### Multidimensional fatigue inventory

The results of the repeated measures analysis indicated a non-significant Time X Condition interaction after V̇O_2max_ in any hypoxic training protocol for general fatigue (SH-TL: *F*_2,18_ = 1.448, *ns*, *η^2^_p_* = .088, SL-TH: *F*_2,18_ = 1.095, *ns*, *η^2^_p_* = .064 & control group *F*_2,18_ = .038, *ns*, *η^2^p* = .003), for physical fatigue (SL-TH: *F*_2,18_ = .004, *ns*, *η^2^_p_* = .000, SH-TL: *F*_2,18_ = .340, *ns*, *η^2^_p_* = .022 & control group: *F*_2,18_ = 3.974, *p* *=* *.*068, *η^2^_p_* = .234), reduced activation (SLTH: *F*_2,18_ = .887, *ns*, *η^2^_p_* = .053, SH-TL: *F*_2,18_ = 478, *ns*, *η^2^_p_* = .031 & control group: *F*_2,18_ = .149, *ns*, *η^2^_p_* = .011), reduced motivation (SL-TH: *F*_2,18_ = 1.205, *ns*, *η^2^_p_* = .070, SH-TL: *F*_2,18_ = .004, *ns*, *η^2^_p_* = .000 & control group: *F*_2,18_ = .020, *ns*, *η^2^_p_* = .891) and mental fatigue (SL-TH: *F*_2,18_ = 2.977, *ns*, *η^2^_p_* = .157, SH-TL: *F*_2,18_ = .680, *ns*, *η^2^_p_* = .043 & control group: *F*_2,18_ = .913, *ns*, *η^2^_p_* = .066).

**Figure 2:**
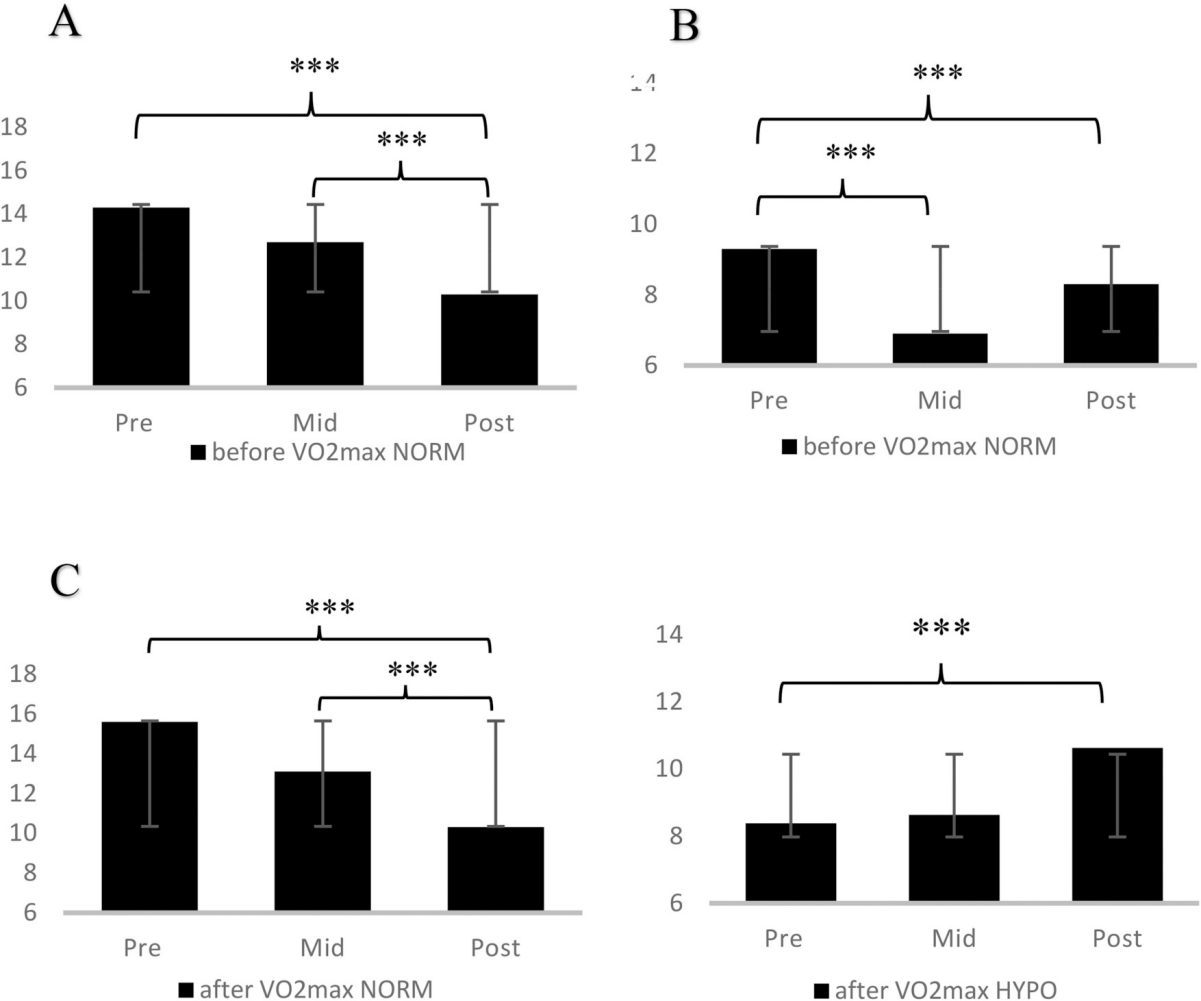
Energy **(A)**, tension **(B)** in SL-TH before V̇O_2max_ in normoxia, energy **(C)** in SL-TH after V̇O_2max_ in normoxia and tension **(D)** in SL-TH after V̇O_2max_ in hypoxia. *** *p* < .001.

**Figure 3:**
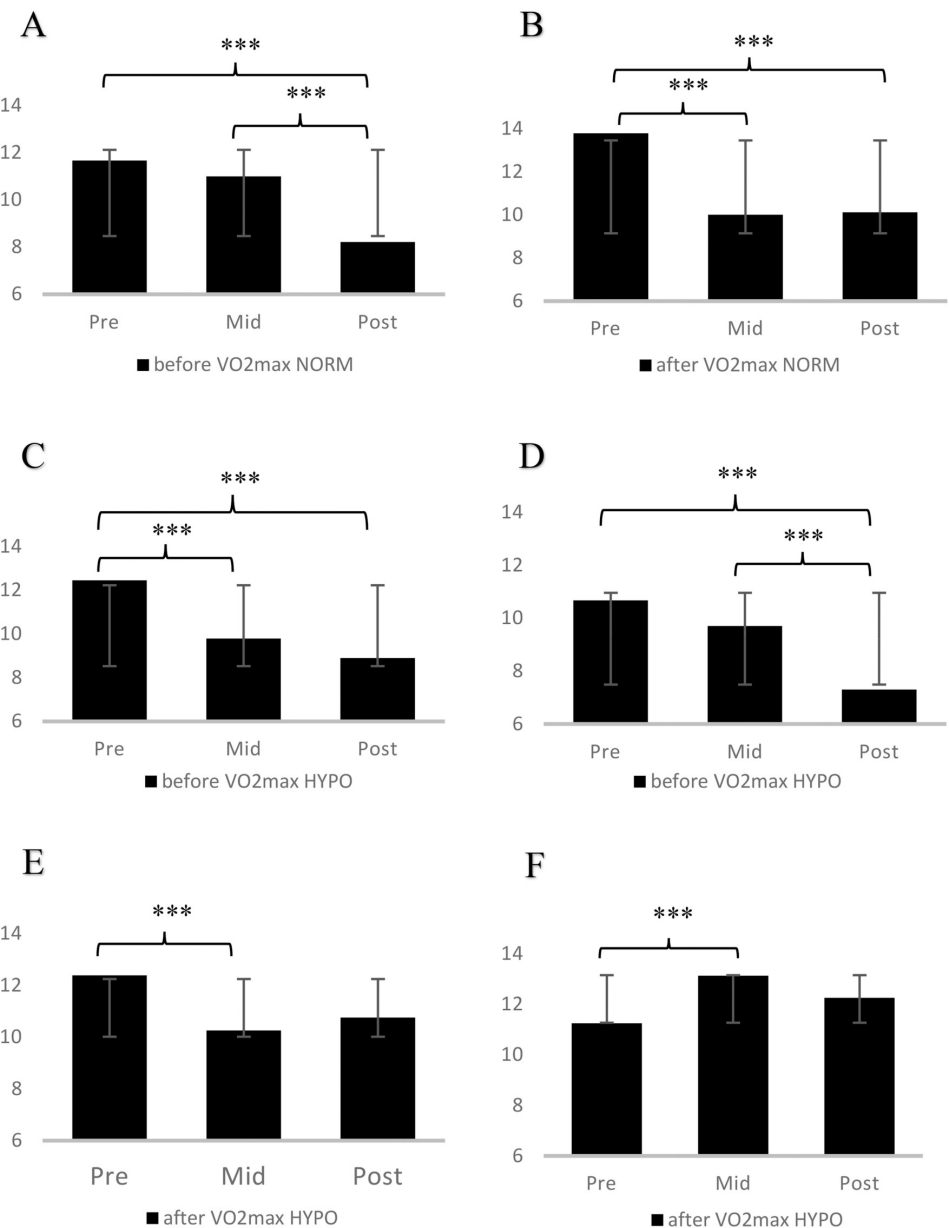
Tiredness **(A)** in SH-TL before V̇O_2max_ and calmness **(B)** in SH-TL after V̇O_2max_ in normoxia, tiredness **(C)**, tension **(D)** in SH-TL before V̇O_2max_ and calmness **(E)**, general fatigue **(F)** in SH-TL after V̇O_2max_ in hypoxia.

Tables 1, 2 present the descriptive statistics (*M* *±* *SD*) to provide a clear overview of the data for the ADACL and MFI subscales, respectively. To avoid excessive detail and maintain readability, significance indicators were not included in the Tables 1, 2. Instead, statistically significant differences are placed in the Figures 2, 3 and explained in the text, with corresponding annotations provided in the figure legends for clarity.

**Table 1:** Descriptive statistics (mean ± SD) of the activation-deactivation adjective check -list (ADACL) subscales in the experimental conditions.

ADACL subscales	Training protocol	V̇O_2max_	Normoxia		Hypoxia	
			Pre	Post	Pre	Post
			M ± SD	M ± SD	M ± SD	M ± SD
Energy	SL-TH	before	14.30 ± 3.30	10.30 ± 4.00	13.90 ± 3.54	10.75 ± 3.06
		After	15.60 ± 1.65	10.30 ± 3.40	13.50 ± 2.93	12.00 ± 2.51
	SH-TL	before	14.55 ± 2.96	13.00 ± 2.35	12.00 ± 3.10	12.44 ± 2.88
		After	14.33 ± 2.65	13.89 ± 2.80	14.89 ± 1.55	13.25 ± 2.38
	Control	before	16.00 ± 3.20	13.13 ± 5.38	15.25 ± 2.87	13.50 ± 4.01
		after	14.50 ± 4.66	14.63 ± 4.87	15.50 ± 3.42	13.00 ± 5.37
Tiredness	SL-TH	before	10.50 ± 1.78	10.90 ± 1.37	11.13 ± 1.64	10.13 ± 1.81
		after	12.10 ± 1.73	12.70 ± 1.57	12.88 ± 1.25	12.50 ± 1.77
	SH-TL	before	11.67 ± 2.55	8.22 ± 1.79	12.44 ± 1.59	8.89 ± 1.54
		after	12.00 ± 2.12	10.22 ± 3.15	11.33 ± 2.83	10.25 ± 2.82
	Control	before	10.75 ± 2.25	12.25 ± 2.82	10.38 ± 2.88	10.63 ± 2.72
		after	11.88 ± 2.17	10.75 ± 2.96	12.25 ± 2.81	11.50 ± 3.74
Tension	SL-TH	before	9.30 ± 2.21	8.30 ± 2.21	8.25 ± 1.75	9.13 ± 4.02
		after	9.60 ± 2.72	9.70 ± 2.83	8.40 ± 2.62	10.63 ± 4.03
	SH-TL	before	10.78 ± 3.60	7.44 ± 1.81	10.67 ± 3.24	7.33 ± 3.20
		after	10.67 ± 3.39	8.78 ± 3.03	10.25 ± 2.92	8.63 ± 3.70
	Control	before	9.38 ± 3.42	9.63 ± 4.60	8.38 ± 3.86	8.88 ± 4.67
		after	7.88 ± 3.04	9.00 ± 4.31	9.38 ± 4.07	9.00 ± 4.50
Calmness	SL-TH	before	10.90 ± 2.56	11.10 ± 3.45	10.63 ± 3.34	11.50 ± 3.89
		after	11.40 ± 3.44	11.20 ± 3.47	9.50 ± 3.69	11.13 ± 4.32
	SH-TL	before	12.78 ± 3.15	10.67 ± 3.08	12.44 ± 2.74	10.78 ± 2.68
		after	13.78 ± 2.44	10.11 ± 3.44	12.38 ± 2.77	10.75 ± 3.15
	Control	before	14.25 ± 2.25	11.63 ± 4.37	12.63 ± 3.42	13.00 ± 5.37
		after	13.63 ± 3.29	11.50 ± 4.78	11.75 ± 3.58	11.88 ± 4.49

**Table 2:** Descriptive statistics (mean  SD) of the multidimensional fatigue inventory (MFI) subscales in the experimental conditions.

MFI subscales	Training protocol	Normoxia		Hypoxia	
		Pre M ± SD	Post M ± SD	Pre M ± SD	Post M ± SD
General fatigue	SL-TH	11.90 ± 1.66	11.20 ± 1.40	12.90 ± 1.73	11.13 ± 0.83
	SH-TL	11.67 ± 1.41	11.44 ± 1.02	11.25 ± 1.49	12.25 ± 1.58
	Control	12.71 ± 1.38	12.57 ± 1.27	11.25 ± 2.05	10.88 ± 1.73
Physical fatigue	SL-TH	11.50 ± 2.32	11.70 ± 1.34	11.90 ± 1.25	12.00 ± 2.33
	SH-TL	12.11 ± 1.05	12.22 ± 1.39	11.88 ± 0.64	11.63 ± 0.92
	Control	11.86 ± 1.68	12.57 ± 0.79	12.38 ± 1.19	11.00 ± 1.77
Reduced activation	SL-TH	13.10 ± 2.08	12.50 ± 2.27	12.00 ± 1.70	12.63 ± 1.92
	SH-TL	12.11 ± 1.27	12.11 ± 1.10	12.25 ± 2.25	11.63 ± 1.18
	Control	12.43 ± 1.99	12.00 ± 1.00	11.88 ± 0.83	11.88 ± 2.10
Reduced motivation	SL-TH	11.40 ± 2.54	10.60 ± 1.96	10.75 ± 1.16	11.13 ± 2.23
	SH-TL	10.89 ± 2.85	10.44 ± 2.13	11.00 ± 1.10	10.62 ± 1.69
	Control	10.71 ± 1.11	10.71 ± 1.70	10.25 ± 1.75	10.13 ± 2.36
Mental fatigue	SL-TH	10.60 ± 1.51	10.90 ± 1.52	11.50 ± 0.53	10.63 ± 2.00
	SH-TL	11.22 ± 1.56	10.89 ± 2.67	10.63 ± 1.77	11.63 ± 2.00
	Control	11.71 ± 0.95	10.57 ± 1.13	11.00 ± 1.60	10.75 ± 1.49

Exercise effects on psychological responses during normoxia and hypoxia before and after V̇O_2max._

### SL-TH training protocol

In the normoxic condition, examining the differences between the emotional response across the three-time measures (pre-, mid-, post-test), significant differences revealed before V̇O_2max_ on energy (*F*_3,30_ = 6.479, *p* < .05, *η^2^_p_* = .618) and tension (*F*_3,30_ = 4.457, *p* = .05, *η^2^_p_* = .527). In addition, significant differences indicated after V̇O_2max_ on energy (*F*_3,30_ = 8.924, *p* < .05, *η^2^_p_* = .690). The energy before and after V̇O_2max_ was significantly higher on the pre-test and mid-test compared to the post-test (*p* < .001). The tension decreased in the mid- and post-test compared to the pre-test (*p* < .001). In the hypoxic condition, the results revealed a significant difference on the tension (*F*_3,30_ = 11.039, *p* < .05, *η^2^_p_* = .789), as the tension significantly increased in the post-test compared to the pre-test (*p* < .001). The means, standard deviations, and the level (*p*-values) of statistical significance of the exercise effects in SL-TH participants are presented in Figure 2.

### SH-TL training protocol

In the normoxic condition, examining the differences in participants' emotional response across the three-time measures (pre-, mid-, post-test), the analysis revealed significant differences in tiredness (*F _3,27_* = 11.117, *p* < .05, *η^2^_p_* = .761) before V̇O_2max_ and in calmness (*F*_3,27_ = 11.554, *p* < .05, *η^2^_p_* = .768) after V̇O_2max_. The tiredness was higher on the pre- and mid-test compared to the post-test, as well as the calmness after V̇O_2max_ was higher on pre-test compared to the mid-test (*p* < .001) and the post-test (*p* < .001).

In the hypoxic condition, the tiredness (*F _3,27_* = 6.933, *p* < .05, *η^2^_p_* = .665) and the tension (*F_3,27_* = 7.668, *p* < .05, *η^2^_p_* = .687) showed significant differences over the time before V̇O_2max_. There was a significant decrease in tiredness from pre-test to mid-test (*p* < .001) and to post-test (*p* < .001). In addition, there was a significant decrease in tension from pre- to post-test (*p* < .001), and also from mid- to post-test (*p* < .001). After V̇O_2max,_ the calmness and the general fatigue indicated significant differences over time. Specifically, there was a significant decrease in calmness from pre- to mid-test (*p* < .001), and a significant increase in general fatigue from pre- to mid-test (*p* < .001). The means, standard deviations, and the level (*p*-values) of statistical significance of the exercise effects in SH-TL participants are presented in Figure 3.

## Discussion

The main findings of the current study indicate that a severe lack of oxygen during intense exercise leads to negative emotions and decreased energy levels immediately after exercise, regardless of whether participants are in normal or oxygen-deprived conditions. Specifically, the Sleep Low-Train High (SL-TH) method resulted in lower energy expenditure after maximum oxygen uptake tests, under both normal and low-oxygen settings, than the Sleep High-Train Low (SH-TL) protocol and the control group. However, SH-TL and control participants did not show significant emotional changes. The observed emotional shifts are linked to reduced oxygen conditions, consistent with existing evidence that extreme environments influence human mood (12, 39–41), and physical activity in low-oxygen settings [Seo, Fennell, Burns, Pollock, Gunstad, et al., 2015 (26, 27, 42)]. Taken together, these results suggest that hypoxia serves as an additional physiological stressor, potentially heightening the body’s stress response and leading to prolonged high levels of stress hormones (e.g., cortisol) and sympathetic nervous system dominance. This sustained physiological stress can overwhelm adaptive mechanisms, thereby directly contributing to the development of emotional exhaustion. Concluding, these mood changes could be linked to specific physiological mechanisms such as altered oxygen-transport or shifts in neuromodulators. The decrease in oxygen availability in hypoxia may affect the central nervous system, leading to changes in subjective feelings of fatigue and mood due to the body's adaptive responses.

Previous findings have shown that at high altitudes, above 4,000 meters, increased symptoms of depression, anger, fatigue, euphoria, irritability, and hostility have been observed (5, 43). However, at lower altitudes, the effect of hypoxia on emotional responses remains unclear, as many factors influence them. These factors include acclimatization (23, 29), prolonged exposure to hypoxia (44), effort intensity (44), interpersonal differences (44), participants' personality (45, 46, 60), subjective experience (47, 48, 61), and methodological limitations (5). Understanding these variations can be further enriched by linking them to motivational theories and how extreme environments alter humans' psychological responses. For instance, according to self-determination theory, a decrease in perceived autonomy or competence in challenging conditions, as participants might experience during hypoxia, could result in negative emotional outcomes. In other words, an individual's underlying motivation can influence their emotional response to hypoxia. Highly motivated individuals may be better equipped to sustain effort and regulate negative feelings, buffering the psychological impact of oxygen deprivation. Further to the above, the psychological mechanisms behind these emotional changes may also be explained by affect regulation and cognitive-energy models. Affect regulation suggests that emotional responses are influenced by an individual's capacity to manage emotional states, which may be compromised in hypoxic conditions. Additionally, cognitive-energy models suggest that decreases in oxygen availability may impair cognitive functions, leading to heightened emotional responses. By anchoring these responses to their underlying motivational constructs and psychological mechanisms, we can gain a more comprehensive understanding of the interplay between physical challenges and emotional states. The key point is that integrating psychological monitoring, like affect and mood responses, is essential for athletes and individuals exposed to hypoxic environments to ensure.

The impact of exercise on emotional responses, particularly under varying environmental conditions, including hypoxia and normoxia, remains a topic of debate. While some research suggests that immediate positive or negative mood effects occur regardless of the environment (42), the intensity of exercise in hypoxic conditions appears to be crucial for its impact. Specifically, mood improves only at low to moderate exercise intensities (40%–60% V̇O_2max_) in hypoxia (26, 27). Other studies have found no significant mood changes during hypoxic training (7). These conflicting findings highlight the complexity of the relationship between exercise and mood in different environments. Our research contributes to this discourse by indicating that certain training protocols elicit distinct emotional responses: SL-TH training decreases energy in normoxia and increases tension in hypoxia. In contrast, the SH-TL protocol elicited more positive emotional outcomes in both environments, with decreases in negative emotions and moderate increases in positive emotions, consistent with prior studies (28). Furthermore, many studies support the notion that the SH-TL protocol may also enhance performance at sea level, although some evidence suggests otherwise (49, 55, 56). Additionally, we observed that while general fatigue increased midway through the test in hypoxia, it returned to baseline levels by the end of the test. In summary, the SH-TL protocol emerges as a promising strategy for facilitating emotional adjustment and potentially improving performance at sea level. Integrating this with broader psychological theories, the concept of “environmental stressors” can help frame our hypoxia findings within established theories of mood. Hypoxic conditions, as an environmental stressor, may interact with place affect, impacting mood outcomes during exercise, supported by evidence from environmental psychology (50). Future studies could further explore these connections to expand the understanding of exercise in varying environments.

Further to the above, previous research findings have shown that perceived fatigue increases in a hypoxic environment (25, 51, 52), suggesting that it is an important factor as it affects and regulates both functionality and physical capacity during exercise. However, despite hypoxia-induced fatigue observed in other contexts, participants in the SH-TL training protocol reported no significant effects on fatigue, maintaining baseline levels by the study's end. This could be attributed to the positive effect of exercise on the feeling of fatigue despite the presence of hypoxia (20, 21).

### Practical implications

The results of the present study demonstrate that exposure to hypoxia and exercise affects emotional responses (53). The SH-TL training protocol appears to be more suitable for maintaining mood during the training process compared to the SL-TH training protocol, as it seems to be a better strategy for acclimatization in both hypoxic and normoxic conditions. Physiologically, the SH-TL protocol is linked to improved ventilatory efficiency, which athletes may recognize as smoother and less laborious breathing during exercise, contributing to better mood states. To enhance the reliability of these findings, the study anchored mood assessments using established affect-assessment scales, such as the ADACL and MFI. By referencing these validated tools, readers can more accurately assess the reliability of the mood outcomes presented. Further investigation is needed to identify the most appropriate hypoxic acclimatization strategies that can mitigate the emotional changes associated with hypoxic training and the unpleasant post-exercise emotions induced by hypoxia exposure.

Understanding emotional responses during exercise in hypoxic and normoxic conditions provides valuable insights for designing effective hypoxic training protocols, helping to improve athletes' performance. Implementing the most effective training regimen, such as SH-TL, can help prevent cognitive and performance losses associated with suboptimal acclimatization. By highlighting the decision as a choice between avoidable fatigue and improved readiness, the urgency to select the correct protocol is underscored. This approach is not only helpful for athletes to avoid overtraining and maximize their performance by optimizing their training, but it may also be beneficial for soldiers by preventing emotional exhaustion and enhancing mental toughness. Furthermore, it may benefit patients with chronic diseases, such as COPD, by optimizing their rehabilitation program and improving their overall well-being. To make the relevance of SH-TL even more apparent in clinical contexts, referencing familiar rehabilitation endpoints, such as improvements in short-walk distance or reductions in dyspnea scores, can bridge the gap between athletic and clinical applications.

### Study limitations and methodological considerations

While the present study provides important insights into the independent and combined effects of two different hypoxic training protocols on psychological status, certain limitations should be taken into account when interpreting the obtained outcomes. A potential limitation of the study is that all measurements were undertaken in laboratory conditions, limiting the generalizability of the conclusions. This is particularly relevant when considering real-world settings such as elite training camps and alpine rescue operations, where hypoxic conditions frequently occur. In these environments, factors like sleep quality, hydration level, and diet may significantly influence psychological responses to hypoxia, yet they were not examined in this study. Finally, an important parameter that should be considered is that participants provided their responses to the questionnaires in normoxic conditions. Furthermore, the generalizability of the present findings is limited due to the sample characteristics, which it does not include athletes, female participants, or older adults. It is important to note that the relatively small sample size, the male-only cohort, and the limited protocol duration consist study limitations. Moreover, the absence of physiological parameters and biomarkers represents a methodological constraint, as it restricts the capacity to interpret the underlying biological mechanisms associated with emotional responses to hypoxic training protocols. These factors may impact the generalizability of the findings, and future research is encouraged to explore these variables in more diverse and larger populations.

Future research is proposed to examine alternative hypoxic training protocols, specifically emphasizing the promising candidates such as intermittent normobaric and continuous hypobaric modalities. Focusing on these could provide a more comprehensive understanding of the effects of hypoxia on emotional responses and determine which protocols could be more effective not only in performance but also in emotional responses. It is recommended that future research include women and evaluate additional emotional characteristics to provide a more comprehensive picture of their emotional state. Finally, it would be beneficial for the participants to provide their responses to questionnaires in hypoxic conditions to gain a more accurate understanding of the effect of hypoxia on participants' emotional responses. Despite the aforementioned constraints, the research findings offer valuable insights regarding the emotional dynamics elicited during human exposure to hypoxic exercise conditions.

## Data Availability

The raw data supporting the conclusions of this article will be made available by the authors, without undue reservation.
